# Multiple Autoimmune Syndrome With Alopecia Universalis and Immune Thrombocytopenic Purpura

**DOI:** 10.7759/cureus.13033

**Published:** 2021-01-31

**Authors:** Turki A Alwasaidi, Waleed Mustafa, Haitham Osman, Abdulqader Al-Hebshi, Asma Alfetayeh

**Affiliations:** 1 Hematology Division/Internal Medicine Department, Prince Mohammed Bin Abdulaziz Hospital - Ministry of National Guard Health Affairs, Madinah, SAU; 2 Internal Medicine, Taibah University, Madinah, SAU; 3 Hematology, University Hospital Limerick, Limerick, IRL; 4 Pediatrics-Hematology Oncology, Prince Mohammed Bin Abdulaziz Hospital, Madinah, SAU; 5 Pediatrics, King Saud Bin Abdulaziz University for Health Sciences, Riyadh, SAU; 6 Hematology, Prince Mohammed Bin Abdulaziz Hospital, Madinah, SAU

**Keywords:** alopecia universalis, multiple autoimmune syndrome ( mas ), immune thrombocytopenia purpura, itp, autoimmune hypothyroidism

## Abstract

Multiple autoimmune syndrome (MAS) is a condition characterized by three or more autoimmune disorders in the same individual. The development of MAS involves genetic, immunological, and infectious factors. Here we report a case of a 40-year-old man who presented with four autoimmune diseases, namely autoimmune hypothyroidism, alopecia universalis, celiac disease, and immune thrombocytopenic purpura (ITP), which leads to a diagnosis of MAS. However, the patient does not fit in any category of MAS classification. In addition to the need for continued surveillance for the development of new autoimmune disease in predisposed patients, this case report suggests an additional fourth category of the classification of MAS that includes autoimmune hypothyroidism, alopecia universalis, celiac disease, and ITP.

## Introduction

Autoimmune diseases are initiated by the loss of immunological tolerance to self-antigens and that makes up, a heterogeneous group of disorders in which multiple alterations in the immune system result in targeting specific organs by immune responses or affect the body systematically [1]. Multiple autoimmune syndrome (MAS) is defined as the occurrence of at least three autoimmune diseases in the same patient [2]. It is further classified into type 1 MAS, type 2 MAS, and type 3 MAS. Celiac disease affects 1% of the general population and is an important autoimmune disease, but it is not included in the classification of MAS. However, dermatitis herpetiformis, which is one of the associated conditions with celiac disease, is one of the conditions that define type 3 MAS. Celiac disease is strongly associated with autoimmune hypothyroidism and Sjögren’s syndrome in 2%-7% and 4.5%-15%, respectively [3,4] and is very less commonly associated with systemic lupus erythematosus (SLE). It is noteworthy that the existence of one autoimmune disease can contribute to the detection and diagnosis of other autoimmune conditions.

## Case presentation

The 40-year-old male patient is known to have hypothyroidism, which was diagnosed two years ago, and currently on thyroxin replacement therapy. The patient was diagnosed with alopecia universalis 12 months ago, when he gradually lost his all body hair over four months. He followed up by a dermatologist who treated him with local steroid injection to maintain his eyebrows hair and mustache. He was also diagnosed with iron deficiency anemia, ferritin was 20.4 ng/ml (normal: 20-360 ng/ml), and with B12 deficiency which was 174 pg/ml (n: 187-883 pg/ml) six months before his presentation and treated with parenteral iron (iron 200mg X 5 doses) and cyanocobalamin (vitamin B12) supplement (B12 1000 mcg X 12 injections).

He presented to the hospital after referral for incidental thrombocytopenia. His platelet count was 40×109/l (discovered three weeks before the presentation) and dropped to 24×109/l in one week. There was no history of bleeding from any site, no petechiae or easy bruising, no preceding febrile illness or flu-like symptoms no joint pain or swellings. No family history of thrombocytopenia or rheumatological disease or use of quinine or diuretics. Social history showed that he was a smoker (11 pack-year history) and he is a non-alcohol consumer.

Physical examinations showed total loss of body hair including the scalp, eyebrows, and mustache. His skin was generally erythematous; no typical malar rash or petechial rash or ecchymosis, no lymphadenopathy, or gingival hyperplasia, no hepatsplenomegaly.

Work up carried out included autoimmune serology which was positive for anti-thyroid peroxidase (TPO), anti-gastric parietal cell, anti-tissue transglutaminase (tTG), and anti-gliadin antibodies. Hepatitis serology, HIV, Helicobacter pylori (H. pylori) stool antigen, and anti-intrinsic factor antibodies were negative. Blood smear showed thrombocytopenia and mild leukocytosis presented as a mild left shift of neutrophilia and toxic granulations.

Because of the combination of iron deficiency and B12 deficiency, the patient underwent gastroscopy with duodenal biopsy which showed no histologic features suggestive of celiac disease or gastritis. As a part of the ITP workup, he underwent bone marrow aspiration that showed normal trilineage and adequate megakaryocytes without infiltration. Cytogenetic analysis showed normal karyotype which indicated peripheral platelet destruction in keeping with the diagnosis of ITP. An abdominal ultrasound revealed no features of chronic liver disease, no hepatomegaly, no splenomegaly or splenic atrophy, no adenopathy. His recent B12 and iron profile were in the normal range as already he received B12 and iron supplements prior to presentation to our hospital.

His thrombocytopenia was initially treated with high dose prednisolone (1 mg/kg). There was a suboptimal response in his platelet count but his alopecia universalis improved partially. Upon tapering the prednisolone dose, his hair fall recures back. After six weeks on steroid, his ITP was labeled as steroid refractory so he went for the second-line treatment which was rituximab 375 mg/m^2^ weekly times X4 with no response in both his platelet count and alopecia universalis. Then he was switched to the 3rd line eltrombopag (thrombopoietin receptor agonist) 75 mg orally once daily along with azathioprine 50 mg oral daily after which his platelet count increased and reached 106x10^9^ for the first time and his facial and scalp hair showed good improvement; he is on current treatment almost for 12 months now. He was continued on parenteral B12 monthly and he was advised to avoid gluten-containing diet to prevent subtotal villous atrophy in the future. Table 1 shows the workup investigations.

**Table 1:** Laboratory investigations. ALT: alanine aminotransferase; AST: aspartate aminotransferase; TSH: thyroid-stimulating hormone; T4: thyroxine; TPO: thyroid peroxidase antibodies; GPA: gastric parietal cell antibody.

Platelet	106 (n: 150-450 ×109/l)
Serum ferritin	20.4 (n: 20-300 ng/ml)
Vitamin B12	210 (n: 187-883 pg/ml)
Serum iron	53 (n: 49-181 mcg/l)
Folate	29.1 (n: 7.9-46.4 nmol/L)
TSH	0.90 (n: 0.35-4.94 mIU/L
Free T4	16.00 (n: 9.00-19.00 pmol/L)
TPO	18.53 (n 1.00-16.00 IU/mL)
Serum calcium	9.7 (n: 8.4-10.2 mg/dl)
ALT	23 (n: 21-72 u/l)
AST	15 (n: 21-72 u/l)
Intrinsic factor antibodies	<2 (n: up to 20 AU/ml)
Anti-tissue transglutaminase IgA (tTG) antibodies	30.93 (n: < 25 IU/ml)
Anti-tissue transglutaminase IgG (tTG) antibodies	7.98 (n: < 25 IU/ml)
GPA antibodies	103.67 (<20 negative - 20.1-24.9 equivocal - >25 positive)
Anti-endomysial IgA antibodies	Titer <1:10 (<1:10 negative - >1:10 positive)
Anti-gliadin IgA antibodies	27.34 (<20 is negative - >20 is positive)
Anti-gliadin IgG antibodies	5.28 (<20 is negative - >20 is positive)

## Discussion

MAS describes the presence of three or more autoimmune disease in one patient. Additionally, it is classified into three types: type 1, type 2, and type 3 (Table 2). This classification may help in uncovering other autoimmune diseases in the same patient [5]. The pathogenesis of MAS is not clearly understood, but many environmental factors in genetically predisposed people are thought to cause disorders of immune responses.

**Table 2:** Classification of multiple autoimmune syndrome. MAS: multiple autoimmune syndrome; ITP: immune thrombocytopenic purpura.

Type 1 MAS	Type 2 MAS	Type 3 MAS
Myasthenia gravis	Sjögren’s syndrome	Sjögren’s syndrome
Thymoma	SLE	Myasthenia gravis and or thymoma
Polymyositis	Rheumatoid arthritis	Addison’s disease
Giant cell myocarditis	Primary biliary cirrhosis	Type 1 diabetes mellitus
	Scleroderma	Autoimmune haemolytic anaemia, pernicious anaemia, ITP
	Autoimmune hypothyroidism	Autoimmune hypothyroidism
		Dermatitis herpetiformis

**Figure 1:**
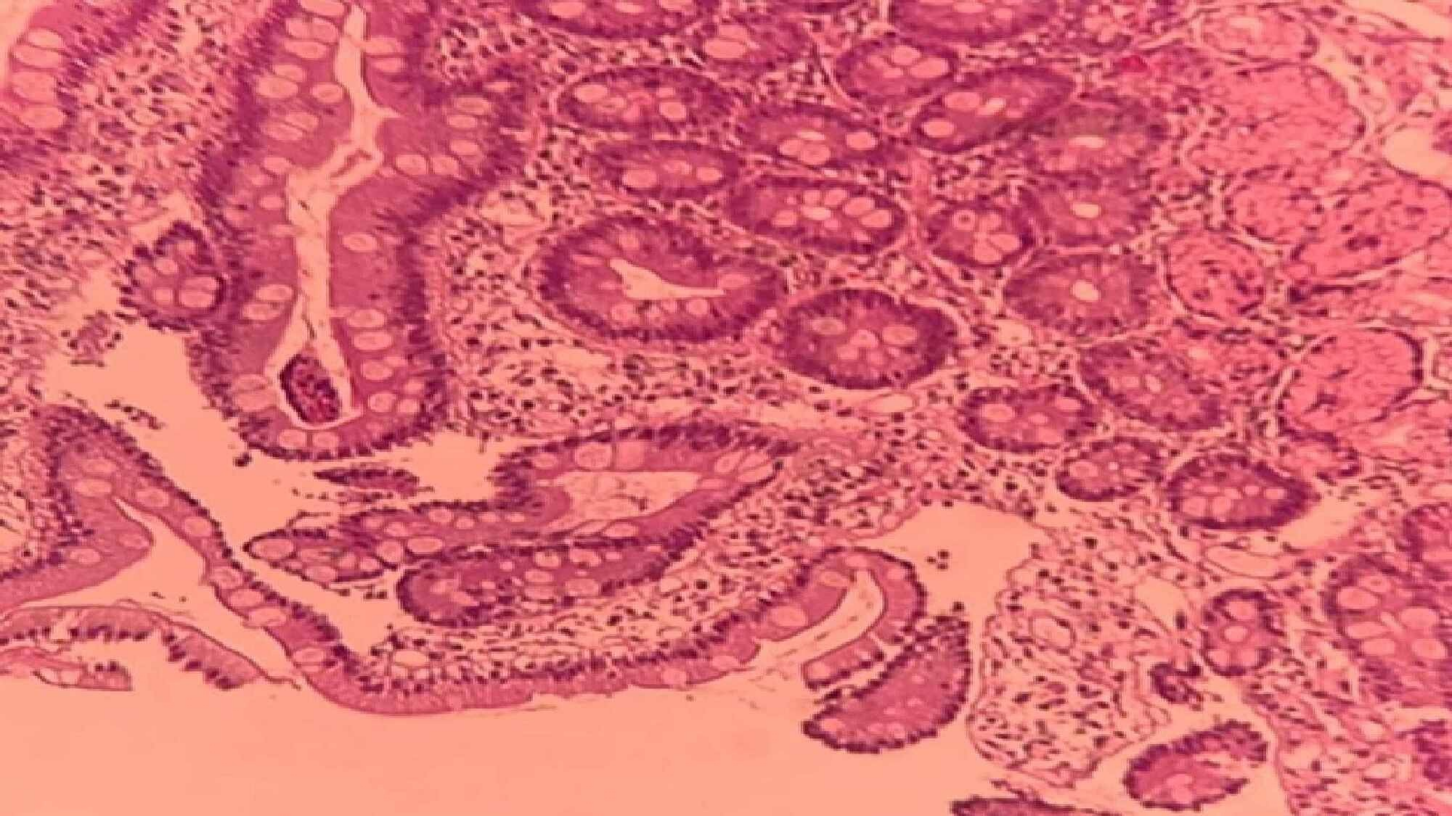
Endoscopic small bowel biopsy of the patient shows normal duodenal histology.

The patient was diagnosed previously with autoimmune hypothyroidism based on positive serology for thyroid peroxidase (TPO) IgG antibodies. These antibodies are considered to be secondary to the thyroid damage inflicted by T cells [6]. As an advanced form of alopecia areata (loss of scalp hair), the diagnosis of AU was clinically made. Alopecia areata is associated with a variety of autoimmune diseases. The strongest association has been with autoimmune thyroid disorders which occurs in 8% to 28%of patients [7,8]. Patients with MAS often have a dermatological condition, usually vitiligo or alopecia areata [9]. Considering the patient's medical history of iron deficiency anemia and B12 deficiency, a serologic evaluation, to detect tissue transglutaminase and gliadin antibodies, was initiated for the diagnosis of celiac disease. Serology was positive for IgA anti-tTG and IgA anti-gliadin antibodies. Most studies describe anti-tTG antibodies as highly sensitive and specific for the diagnosis of celiac disease [10-12]. Upper endoscopy with small bowel biopsy was performed, looking for villous atrophy, but it was negative (Figure 1). Since celiac disease enteropathy can be patchy and missed due to sampling error, numerous biopsies obtained from multiple sites in the mid and distal duodenum are required to detect villous atrophy. One study suggested that a minimum of three biopsies is required to ensure that villous atrophy is detected [13]. Additionally, latent celiac disease can present with normal mucosa [14].

**Figure 2:**
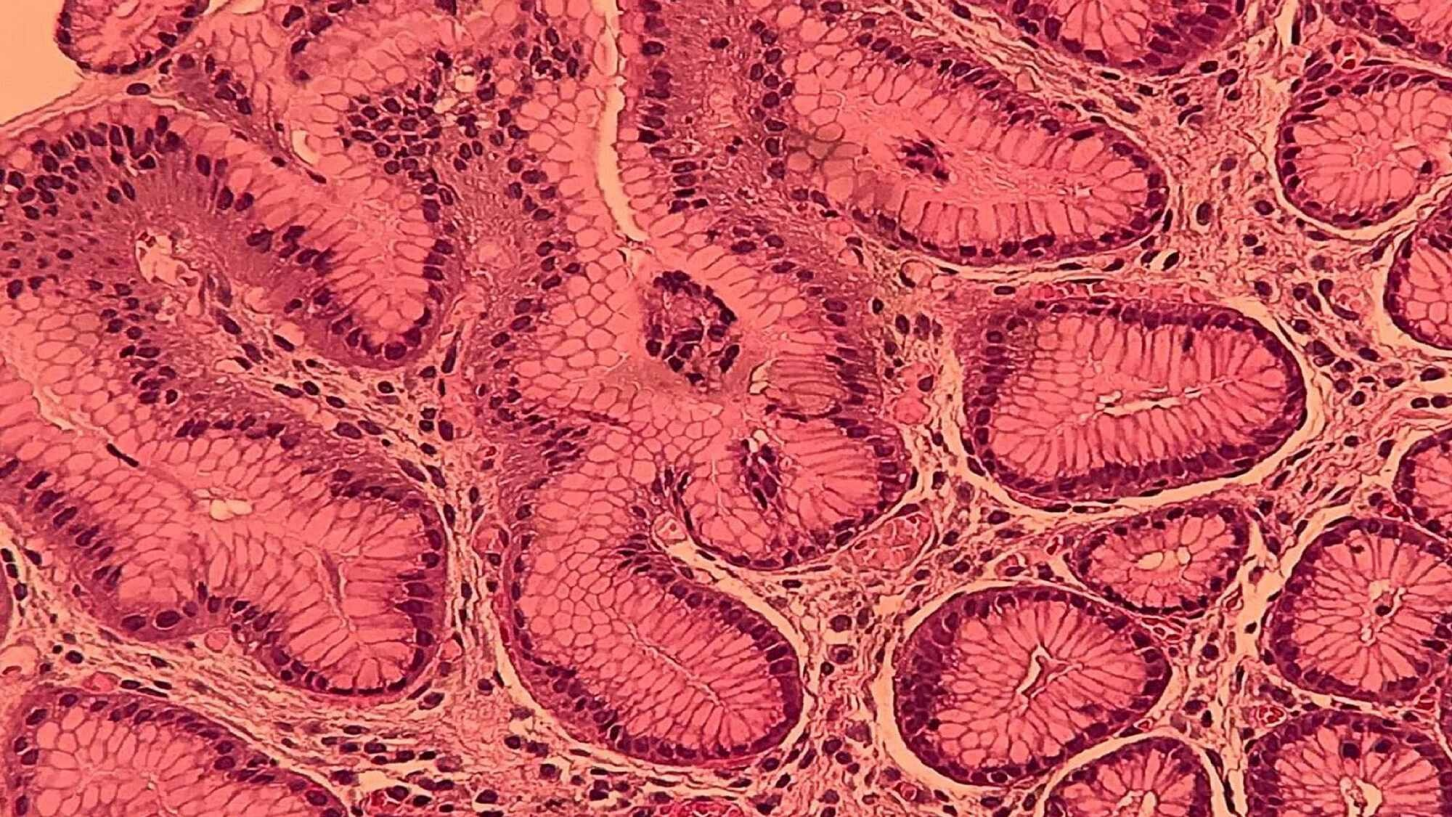
Endoscopic gastric biopsy shows mild non-specific inflammation with intestinal metaplasia. Investigations for H. pylori were negative.

Dermatitis herpetiformis, which is frequently associated with celiac disease, is one of the conditions that classifies MAS into type 3. Although our patient did not develop it, there are associations between dermatitis herpetiformis and autoimmune thyroid disease, pernicious anemia as well as alopecia areata [15]. Furthermore, the gastric biopsy showed mild non-specific inflammation with intestinal (goblet cell type) metaplasia (Figure 2). Autoimmune gastritis, which is distinct from type B gastritis associated with H. pylori, has been linked with the development of gastric intestinal metaplasia [16]. Mild-moderate gastric dysplasia is associated with annual incidences of gastric cancer of 0.6% [17]. Serology for pernicious anemia was conducted and parietal cell antibodies were positive but the intrinsic factor antibodies, which are more specific [18,19], were negative, and hence we could not confirm the diagnosis of pernicious anemia. There is no "gold standard" test that can establish the diagnosis of ITP. The diagnosis depends on the exclusion of other causes of thrombocytopenia. Drug-induced thrombocytopenia, infections such as H. pylori, hypersplenism, liver diseases, myelodysplastic syndromes, thrombotic thrombocytopenic purpura, and congenital thrombocytopenias were all excluded. The presence of the above mention diseases, in one patient, does not fit in any of the three categories of MAS and here we suggest a fourth category that includes autoimmune hypothyroidism, celiac disease, alopecia areata, and ITP (Table 2). 

## Conclusions

Our patient has MAS but does not fit in any category of MAS classification. In addition to the need for continued surveillance for the development of new autoimmune disease in predisposed patients. This case report may suggest an additional fourth category of the classification of MAS that includes autoimmune hypothyroidism, alopecia universalis, celiac disease, and ITP.
